# Supplementary material for: Optimizing irradiation dose for Drosophila melanogaster males to enhance heterospecific Sterile Insect Technique (h-SIT) against Drosophila suzukii
Source: PLoS One. 2025 Jul 3;20(7):e0320546. doi: 10.1371/journal.pone.0320546 (PMC12225833; doi:10.1371/journal.pone.0320546)

PLOS ONE

Advancing Heterospecific Sterile Insect Technique Against Drosophila suzukii: selection of the optimal irradiation dose for D. melanogaster males

--Manuscript Draft--

Manuscript Number:	PONE-D-25-09324
Article Type:	Research Article
Full Title:	Advancing Heterospecific Sterile Insect Technique Against Drosophila suzukii: selection of the optimal irradiation dose for D. melanogaster males
Short Title:	Optimal Irradiation Dose for Heterospecific SIT Against Drosophila suzukii
Corresponding Author:	FLAVIA CERASTI<br>Sapienza University of Rome: Universita degli Studi di Roma La Sapienza<br>Rome, ITALY
Keywords:	Pest control; Reproductive interference; Sterile Insect Technique; Irradiation dose
Abstract:	The spotted-wing drosophila (Drosophila suzukii), a highly invasive agricultural pest, poses significant challenges to fruit production worldwide. Traditional chemical control methods are costly and raise concerns about resistance and environmental sustainability. The Heterospecific Sterile Insect Technique (h-SIT) has emerged as a promising alternative, using sterile heterospecific males (Drosophila melanogaster) to suppress D. suzukii populations through reproductive interference. However, optimizing irradiation doses is critical to balancing male sterility, maintaining biological quality and mating performances. This study aimed to determine the optimal irradiation dose for D. melanogaster males by assessing their sterility, longevity, and courtship behavior following exposure to gamma-ray doses ranging from 80 to 180 Gy. Results showed a significant reduction in fertility across all irradiation doses, with near-complete sterility at 180 Gy. However, longevity decreased with increasing doses, with males irradiated at 160–180 Gy showing a lifespan reduction of up to 50 days compared to controls. Behavioral trials revealed that irradiated D. melanogaster males retained their courtship ability toward D. suzukii females, although males exposed to 160 Gy exhibited reduced courtship activity. These findings highlight that, among the tested doses, 80 Gy emerged as the most effective, preserving male longevity and mating performance while significantly reducing fertility. While 180 Gy achieved the highest sterility, the potential lifespan and courtship behavior trade-offs warrant further evaluation. Future studies should evaluate field performance to refine the balance between sterility, longevity, and mating performances for effective D. suzukii population suppression.
Order of Authors:	FLAVIA CERASTI
	Valentina Mastrantonio
	Alessia Cemmi
	Ilaria Di Sarcina
	Massimo Cristofaro
	Daniele Porretta
Additional Information:	
**Question**	**Response**
**Financial Disclosure**<br><br>Enter a financial disclosure statement that describes the sources of funding for the work included in this submission. Review the submission guidelines for detailed requirements. View published research	Yes

Powered by Editorial Manager® and ProduXion Manager® from Aries Systems Corporation

articles from *PLOS ONE* for specific examples.<br><br>This statement is required for submission and **will appear in the published article** if the submission is accepted. Please make sure it is accurate.<br><br>**Funded studies**<br>Enter a statement with the following details:<br>• Initials of the authors who received each award<br>• Grant numbers awarded to each author<br>• The full name of each funder<br>• URL of each funder website<br>• Did the sponsors or funders play any role in the study design, data collection and analysis, decision to publish, or preparation of the manuscript?<br><br>Did you receive funding for this work?	
Please add funding details.<br>as follow-up to "**Financial Disclosure**<br><br>Enter a financial disclosure statement that describes the sources of funding for the work included in this submission. Review the submission guidelines for detailed requirements. View published research articles from *PLOS ONE* for specific examples.<br><br>This statement is required for submission and **will appear in the published article** if the submission is accepted. Please make sure it is accurate.	FC was funded by PhD resources within PON "RICERCA E INNOVAZIONE" 2014–2020", AZIONE IV.5 "DOTTORATI SU TEMATICHE GREEN, D.M. 1061 10 August 2021.<br>DP was funded by Agritech National Research Center and received funding from the European Union Next-Generation EU (PIANO NAZIONALE DI RIPRESA E RESILIENZA (PNRR) – MISSIONE 4 COMPONENTE 2, INVESTIMENTO 1.4 – D.D. 1032 17 June 2022, CN00000022).

**Funded studies**<br>Enter a statement with the following details:<br>• Initials of the authors who received each award<br>• Grant numbers awarded to each author<br>• The full name of each funder<br>• URL of each funder website<br>• Did the sponsors or funders play any role in the study design, data collection and analysis, decision to publish, or preparation of the manuscript?<br><br>Did you receive funding for this work?"	
Please select the country of your main research funder (please select carefully as in some cases this is used in fee calculation).<br>as follow-up to "**Financial Disclosure**<br><br>Enter a financial disclosure statement that describes the sources of funding for the work included in this submission. Review the submission guidelines for detailed requirements. View published research articles from *PLOS ONE* for specific examples.<br><br>This statement is required for submission and **will appear in the published article** if the submission is accepted. Please make sure it is accurate.<br><br>**Funded studies**<br>Enter a statement with the following details:<br>• Initials of the authors who received each award<br>• Grant numbers awarded to each author<br>• The full name of each funder<br>• URL of each funder website<br>• Did the sponsors or funders play any role in the study design, data collection and analysis, decision to publish, or preparation of the manuscript?<br><br>Did you receive funding for this work?"	ITALY - IT
**Competing Interests**	The authors have declared that no competing interests exist.

Use the instructions below to enter a competing interest statement for this submission. On behalf of all authors, disclose any competing interests that could be perceived to bias this work—acknowledging all financial support and any other relevant financial or non-financial competing interests.<br><br>This statement is required for submission and **will appear in the published article** if the submission is accepted. Please make sure it is accurate and that any funding sources listed in your Funding Information later in the submission form are also declared in your Financial Disclosure statement.<br><br>View published research articles from *PLOS ONE* for specific examples.<br><br>**NO authors have competing interests**<br><br>Enter: *The authors have declared that no competing interests exist.*<br><br>**Authors with competing interests**<br><br>Enter competing interest details beginning with this statement:<br><br>*I have read the journal's policy and the authors of this manuscript have the following competing interests: [insert competing interests here]*<br><br>* typeset	
**Ethics Statement**<br><br>Enter an ethics statement for this submission. This statement is required if the study involved:<br><br>• Human participants<br>• Human specimens or tissue<br>• Vertebrate animals or cephalopods<br>• Vertebrate embryos or tissues	N/A

Powered by Editorial Manager® and ProduXion Manager® from Aries Systems Corporation

• Field research<br><br>Write "N/A" if the submission does not require an ethics statement.<br><br>General guidance is provided below. Consult the submission guidelines for detailed instructions. **Make sure that all information entered here is included in the Methods section of the manuscript.**	

Powered by Editorial Manager® and ProduXion Manager® from Aries Systems Corporation

**Format for specific study types**<br><br>**Human Subject Research (involving human participants and/or tissue)**<br>• Give the name of the institutional review board or ethics committee that approved the study<br>• Include the approval number and/or a statement indicating approval of this research<br>• Indicate the form of consent obtained (written/oral) or the reason that consent was not obtained (e.g. the data were analyzed anonymously)<br><br>**Animal Research (involving vertebrate animals, embryos or tissues)**<br>• Provide the name of the Institutional Animal Care and Use Committee (IACUC) or other relevant ethics board that reviewed the study protocol, and indicate whether they approved this research or granted a formal waiver of ethical approval<br>• Include an approval number if one was obtained<br>• If the study involved *non-human primates*, add *additional details* about animal welfare and steps taken to ameliorate suffering<br>• If anesthesia, euthanasia, or any kind of animal sacrifice is part of the study, include briefly which substances and/or methods were applied<br><br>**Field Research**<br><br>Include the following details if this study involves the collection of plant, animal, or other materials from a natural setting:<br>• Field permit number<br>• Name of the institution or relevant body that granted permission	
**Data Availability**<br><br>Authors are required to make all data underlying the findings described fully available, without restriction, and from the time of publication. PLOS allows rare exceptions to address legal and ethical concerns. See the PLOS Data Policy and FAQ for detailed information.	Yes - all data are fully available without restriction

A Data Availability Statement describing where the data can be found is required at submission. Your answers to this question constitute the Data Availability Statement and **will be published in the article**, if accepted.<br><br>**Important:** Stating 'data available on request from the author' is not sufficient. If your data are only available upon request, select 'No' for the first question and explain your exceptional situation in the text box.<br><br>Do the authors confirm that all data underlying the findings described in their manuscript are fully available without restriction?	
**Describe where the data may be found in full sentences. If you are copying our sample text, replace any instances of XXX with the appropriate details.**<br><br>• If the data are **held or will be held in a public repository**, include URLs, accession numbers or DOIs. If this information will only be available after acceptance, indicate this by ticking the box below. For example: *All XXX files are available from the XXX database (accession number(s) XXX, XXX.).*<br>• If the data are all contained **within the manuscript and/or Supporting Information files**, enter the following: *All relevant data are within the manuscript and its Supporting Information files.*<br>• If neither of these applies but you are able to provide **details of access elsewhere**, with or without limitations, please do so. For example:<br><br>*Data cannot be shared publicly because of [XXX]. Data are available from the XXX Institutional Data Access / Ethics Committee (contact via XXX) for researchers who meet the criteria for access to confidential data.*<br><br>*The data underlying the results presented in the study are available from (include the name of the third party*	Raw data will be available from the authors under request.

and contact information or URL).<br>• This text is appropriate if the data are owned by a third party and authors do not have permission to share the data.<br><br>* typeset	
Additional data availability information:	

Manuscript Click here to access/download;Manuscript;Manuscript.docx

Running title: Optimal Irradiation Dose for Heterospecific SIT Against *Drosophila suzukii*

Advancing Heterospecific Sterile Insect Technique Against *Drosophila suzukii:* selection of the optimal irradiation dose for *D. melanogaster* males

Flavia Cerasti [1*], Valentina Mastrantonio[1], Alessia Cemmi[2], Ilaria Di Sarcina[2], Massimo Cristofaro[3], Daniele Porretta[1]

[1]Department of Environmental Biology, Sapienza University of Rome, Rome, Italy

[2]NUC-IRAD-GAM Laboratory, Italian National Agency for New Technologies, Energy and Sustainable Economic Development (ENEA), Rome, Italy

[3]Biotechnology and Biological Control Agency (BBCA), Rome, Italy

* Corresponding author

E-mail: flavia.cerasti@uniroma1.it (FC)

Abstract

The spotted-wing drosophila (*Drosophila suzukii*), a highly invasive agricultural pest, poses significant challenges to fruit production worldwide. Traditional chemical control methods are costly and raise concerns about resistance and environmental sustainability. The Heterospecific Sterile Insect Technique (h-SIT) has emerged as a promising alternative~~, using~~ sterile heterospecific males (*Drosophila melanogaster*) to suppress *D. suzukii* populations through reproductive interference. ~~However, optimizing irradiation doses is critical to balancing male sterility, maintaining~~ biological

quality and mating performances. This study aimed to determine the optimal irradiation dose for *D. melanogaster* males by assessing ~~their~~ sterility, ~~longevity, and courtship behavior following exposure to gamma-ray doses ranging from 80 to 180 Gy~~. Results showed a significant ~~reduction~~ in fertility across all irradiation doses, with near-complete sterility at 180 Gy. However, longevity decreased with increasing doses, with males irradiated at 160–180 Gy showing a lifespan reduction of up to 50 days compared to controls. ~~Behavioral trials revealed that irradiated~~ *D. melanogaster* males retained their courtship ability toward *D. suzukii* females, although males exposed to 160 Gy exhibited reduced courtship activity. These findings ~~highlight that~~, among the tested doses, 80 Gy ~~emerged as~~ the most effective, preserving male longevity and mating performance while significantly reducing fertility. While 180 Gy ~~achieved~~ the highest sterility, the potential lifespan and courtship behavior trade-offs warrant further evaluation. Future studies should evaluate field performance to refine the balance between sterility, longevity, and mating performances for effective *D. suzukii* population suppression.

1. Introduction

The spotted-wing drosophila (SWD), *Drosophila suzukii* (Matsumura) (Diptera: Drosophilidae) is an invasive agricultural pest native to Southeast Asia (1). Since its first detection in California in 2008, *D. suzukii* has rapidly expanded its geographical distribution in many other states of United States and across the globe, becoming a severe agricultural pest ~~also~~ in Europe, South America, and parts of Africa (2–4). According to recent studies, the economic damage caused by *D. suzukii* in the United States alone reaches hundreds of millions of US dollars annually, ~~and in~~ Europe, similar losses are reported, ~~suffering~~ significant economic damage in their fruit industries (5,6). The rapid spread of *D. suzukii* has been facilitated by its exceptional ability to thrive in diverse environmental conditions, facilitated by its broad temperature tolerance and adaptability to different habitats (1,7). One of the factors contributing to the invasive success of *D. suzukii* is its nutritional versatility. *D. suzukii* can attack a broad range of ripening fruits, including soft-skinned berries and stone fruits ~~with economic~~

~~relevance; then, larval development causes fruit to rot rapidly due to the introduction of rot-type pathogens at the oviposition site (8)~~.

To counter the negative impact of *D. suzukii*, efficient and prompt population control ~~actions~~ are required. Chemical insecticides, especially organophosphates, pyrethroids and spinosyns, ~~were the first and~~ most effective control approach, but this strategy ~~presents~~ multiple challenges (9,10). Insecticides must be applied several times per growing season, due to *D. suzukii* short generation time and larval development inside the fruits (6,9). Thus, repeated exposure, short generation time, and high fecundity have led to metabolic and penetration resistance development to spynosyns and pyrethroids, raising concerns about the environmental sustainability of this approach (11,12). ~~Alternatively,~~ significant research has been devoted ~~to~~ finding sustainable control measures under an Integrated Pest Management approach (13–16).

In recent years, there has been a renewed interest in the use of SIT (Sterile Insect Technique) and the release of sterile heterospecific males (i.e., heterospecific-Sterile Insect Technique) for pest control (17–20). Both approaches can be developed under similar theoretical frameworks. SIT consists ~~in~~ releasing large numbers of sterile males of the target pest species into the environment to mate with conspecific wild females. The unfertile mating between the released sterile males and wild females leads to a gradual decline in the pest population over time (21,22). Contrary to ~~the~~ SIT, in heterospecific SIT, sterile males from closely related species are released to compete with the pest population for mates. The heterospecific SIT leverages reproductive interference, a reproductive interaction between individuals of different animal co-generic species and/or subspecies, which results in fitness costs for one or both the interacting individuals (23–27). It results from incomplete mating barriers between species and can occur at any stage of mate acquisition through different mechanisms, from courtship to mating (24,25,27).

~~The irradiation dose is a key factor for the successful implementation~~ of these approaches, requiring a careful balance between achieving sufficient male sterility and preserving the biological performance of irradiated individuals. Studies on the evaluation of irradiation dose effects on different

insect pests (e.g., *Ceratitis capitata* Wiedemann, *Anopheles arabiensis* Patton, *Aedes aegypti* L., *Aedes albopictus* Skuse) highlighted that an optimal irradiation dose can induce full sterility without significantly compromising the biological qualities of males (28–32). At the same time, improperly calibrated irradiation can lead to males that either retain some level of fertility or exhibit impaired mate-finding abilities (29,30), emphasizing the necessity of refining the irradiation dose to balance sterility and biological fitness.

Our previous studies demonstrated that *Drosophila melanogaster* (Meigen) could be a good candidate for *D. suzukii's* control species into a heterospecific SIT context. The two species have incomplete pre-mating and complete post-mating isolation, and reproductive interference has been documented between them (19). Furthermore, under laboratory conditions, *D. melanogaster* males irradiated at 60 and 80 Gy were able to court and mate with *D. suzukii* females, leading to a significant offspring reduction, although residual fertility has been observed in irradiated males (20). These results provided the first foundation to develop heterospecific SIT against *D. suzukii*.

The aim of this study was to detect the optimal irradiation dose. First, we investigated the effect of 6 different doses from 80 to 180 Gy ~~on the~~ sterility of *D. melanogaster* males and assessed their fertility, through mating trials with *D. melanogaster* females. Second, we investigated the effect of the irradiation on male longevity. Finally, we studied the courtship behavior of irradiated *D. melanogaster* males analyzing the time spent courting *D. suzukii* females in relation to the different irradiation doses administered.

2. Materials and Methods

2.1. Fruit fly colonies and rearing techniques

Drosophila suzukii and *D. melanogaster* used in this study ~~were reared~~ at the Sapienza University of Rome facilities. The colonies are maintained in the BugDorm-4H4545 insect cages (47.5 x 47.5 x 47.5 cm) in a thermostatic chamber at 25 ± 1°C, with a 14:10 hour light-dark cycle and with a

humidity of 70%. The insects were fed with food substrate based on corn flour (84.1% water, 0.7% agar, 3.2% table sugar, 3.6% yeast, 7.2% corn flour, 1.0% soy flour, 0.2% methylparaben dissolved in 25 mL of 70% ethanol) (33). Each week, the substrate was replaced with a fresh one to allow the insects to feed and lay eggs. The previous substrate was labeled and placed in specified containers to allow the development of new individuals within the colony. The colonies had unrestricted access to water due to cotton balls soaked in a sugar-water solution (1:10 ratio), placed on top of the cages.

2.2. *Drosophila melanogaster* males' sterilization and individuals' selection

Sterilization of *D. melanogaster* males was performed at the Calliope gamma irradiation facility at the ENEA Casaccia Research Center (Rome) at different total absorbed doses with a dose rate value of about 130 Gy/h. The Calliope is a pool-type facility equipped with n.25 ^{60}Co radioisotope sources (mean energy 1.25 MeV) in a high volume (7.0 x 6.0 x 3.9 m) shielded cell (34). Males to be sterilized were chosen by checking their emergence from mature pupae into breeding falcons every 30 minutes. In this way, newly emerged males of both species were collected and isolated from females as soon ~~as they were born~~, avoiding unwanted mating before the experiment. The virgin males collected were placed in separate cages by species~~, and after~~ 72-96 hours, ~~they were taken to the irradiation center for sterilization.~~

2.3. Irradiation effect on *D. melanogaster* male' sterility

~~To evaluate the degree of sterility achieved by *D. melanogaster* males after irradiation, we performed mating experiments between irradiated *D. melanogaster* males and fertile *D. melanogaster* females. Seven treatments were set up, the control using non-irradiated individuals, and six treatments relating to the following irradiation doses: 80 Gy, 100 Gy, 120 Gy, 140 Gy, 160 Gy, and 180 Gy. *D. melanogaster* males were selected and irradiated as previously described.~~ Afterward, ~~we placed~~ five

sterilized *D. melanogaster* males and five virgin *D. melanogaster* females inside 50 ml falcon tubes containing food substrate; we adopted the same procedure for the control individuals. We assessed the experiment in a thermostatic chamber at 25 ± 1°C, with a 14:10 hour light-dark cycle and with a humidity of 70%. After six days, we removed the adult individuals and awaited the emergence of the newborns. We counted and noted newly emerged adults daily. We performed five replicates for each condition.

2.4. Irradiation effect on *D. melanogaster* male' longevity

To evaluate the effect of irradiation on the survival of *D. melanogaster* males, we compared the average lifespan between irradiated and non-irradiated *D. melanogaster* males. We selected 72-96-hour-old *D. melanogaster* males that were irradiated as described in the previous paragraph at the following doses: 80 Gy, 100 Gy, 120 Gy, 140 Gy, 160 Gy, 180 Gy. For each dose, we set up an experimental cage (30 x 30 x 30 cm) with 20 individuals each. Two other cages were set up as controls, in which we placed non-irradiated individuals: one cage was called "home control" with individuals maintained at constant conditions of the thermostatic chamber, and one cage called "trip control", with individuals that we transported to the ENEA Calliope–facility, but outside of the irradiation unit. The "trip control" allowed us to evaluate if the transport could induce an impact on the longevity of the individuals. For all conditions, we carried out mortality checks every day until all the individuals died.

2.5. Irradiation effect on *D. melanogaster* male' courtship behavior

We conducted courtship experiments to evaluate the time spent courting *D. suzukii* females by *D. suzukii* and irradiated *D. melanogaster* males and to assess potential differences in the courtship time in relation to the administered irradiation doses. We selected individuals following the methods of the previous analyses. In this experiment, we irradiated 72-96 hours old *D. melanogaster* males only

at 80 Gy, 160 Gy and 180 Gy. We did not consider irradiations at 100, 120, and 140 Gy because they did not lead to significant differences in terms of sterility and longevity (see Results section).

We set up “no-choice” and “choice” experimental trials: in the “no-choice” condition, we placed one *D. suzukii* female with homospecific or heterospecific male into a falcon (15 mL) and analyzed the male courtship time. Specifically, we analyzed: - the courtship time of *D. suzukii* male with a *D. suzukii* female; - the courtship time of *D. melanogaster* male irradiated at 80, 160 and 180 Gy with a *D. suzukii* female. In the “choice” conditions, we placed one *D. suzukii* female with two homospecific or heterospecific males into a falcon (15 mL) to evaluate the male's courtship time. Specifically, we analyzed: - the courtship time of two no-irradiated *D. suzukii* males with a *D. suzukii* female; - the courtship time of one no-irradiated *D. suzukii* male and one *D. melanogaster* male irradiated at 80, 160 and 180 Gy with a *D. suzukii* female. For the observation of behaviors between two *D. suzukii* males, due to their morphological similarity, the videos were analyzed at reduced playback speed to track the individuals and annotate their behaviors accurately. Conversely, in the second condition involving heterospecific males, the two species were distinguishable: *D. suzukii* males possess characteristic black spots on their wings (hence the name "spotted-wing drosophila"), which are absent in *D. melanogaster* males. For all conditions, following a 5-minute acclimation period, we recorded the individual's behavior for 10 minutes using an Olympus Tough TG-6 camera. After recording, we analyzed the videos using the Boris software (Behavioral Observation Research Interactive Software), taking into account the courtship elements such as orientation, touch, wing scissoring, wing spreading, and copulation attempt (35,36). We carried out 20 replications for each trial to ensure the robustness of our data.

2.6. Data analysis

In the sterility experiment, the sterility degree achieved by *D. melanogaster* males was evaluated for each experimental condition, applying a GLM model (generalized linear model with negative

binomial distribution), and the model family was selected comparing the AIC and BIC estimators and the likelihood ratio test. We performed Tukey's multiple comparison test as a post hoc test using the *'multcomp'* package (37). The average percentage of residual fertility in each condition was obtained by calculating the percentage reduction of each replicate in a specific condition compared to the mean of offspring born in the control condition (i.e. 100% fertility) and calculating the mean (± SE) of the percentages obtained in each replicate.

To evaluate the effect of the irradiation dose on the longevity of *D. melanogaster* males, survival distributions of the different *D. melanogaster* groups ('Control cages', 'Control trip', '80 Gy', '100 Gy', '120 Gy', '140 Gy', '160 Gy', '180 Gy') were computed using the Kaplan-Meier method with the '*survival'* package and the differences between survival distributions were estimated using the Log-Rank Test with the *'survminer'* package (38,39).

In the courtship experiment, to compare the courtship time of *D. suzukii* and *D. melanogaster* males in "no-choice" condition, we used a GLM model (generalized linear model with negative binomial distribution), selecting the model family based on the AIC and BIC estimators and the likelihood ratio test. We performed Tukey's multiple comparison test as a post hoc test. In the "choice" condition, we used a GLM model (generalized linear model with negative binomial distribution) to compare the average courtship time of males. Then, we compared the average courtship time of the two males in the same condition using the nonparametric statistical Wilcoxon Signed Rank test using the *'dplyr'* package (40). All analyses were carried out using R Software version 3.6.2. (41).

3. Results

3.1. *Drosophila melanogaster* males' sterilization

We assessed the sterility of *D. melanogaster* males by mating them with *D. melanogaster* females at irradiation doses of 80, 100, 120, 160, and 180 Gy, with a non-irradiated control group for

comparison. The mean number of the emerged adults in the control condition was 171.8 (± 24.47) (mean ± SE) (Table 1). A significant emergence reduction was observed at all irradiation doses. At the 80 Gy irradiation condition, the mean number of emerged adults was 29.2 (± 9.25), while at 180 Gy irradiation, it dropped to 0.8 (± 0.58) (Table 1; Fig 1). The GLM model showed a significant effect of the male irradiation dose on the number of offspring produced by *D. melanogaster* females (Table 2). Tukey's multiple comparison test showed a significant offspring reduction from the control condition to all irradiation conditions, i.e. 80 Gy (z = 4.253, p = <0.001), 100 Gy (z = 5.183, p = <0.001), 120 Gy (z = 5.634, p = <0.001), 140 Gy (z = 6.660, p = <0.001), 160 Gy (z = 7.194, p = <0.001) and 180 Gy (z = 8.317, p = <0.001). There were also significant differences between the 80 Gy irradiation dose and 160 Gy (z = 3.497, p= 0.008) and 180 Gy (z = 5.535, p= <0.001) doses. The 180 Gy dose showed significant differences with 80 Gy (see above), 100 Gy (z = -4.886, p= <0.001), 120 Gy (z = -4.559, p= <0.001) and 140 Gy (z = -3.761, p= 0.003) doses, but not with 160 Gy. We did not observe significant differences between 80, 100, 120 and 140 Gy (Fig 1).

Table 1. The mean number of the emerged *D. melanogaster* adults. Mean number (±SE) of emerged adults and average percentage (±SE) of the residual fertility at the different treatment doses (Gy).

Treatment Dose (Gy)	Mean number of emerged adults (±SE)	Average percentage (±SE) of residual fertility
0 Gy	171.8 (± 24.47)	100 %
80 Gy	29.2 (± 9.25)	17 % (± 5.38)
100 Gy	23.5 (± 5.52)	11.29 % (± 3.45)
120 Gy	19.5 (± 2.25)	9.19 % (± 2.38)
140 Gy	11.75 (± 2.66)	5.59 % (± 1.73)

160 Gy	5.25 (± 2.39)	3.05 % (± 24.47)
180 Gy	0.8 (± 0.58)	0.47 % (± 0.34)

Table 2. Irradiation effect on *D. melanogaster* male' sterility. GLM model values are shown. Values in boldface indicate significant differences.

**Fixed Effects**	**Estimate**	**±SE**	**z Value**	***p*-Value**
(Intercept)	2.9653	0.3051	9,718	**< 2e-16**
120 Gy	-0.2053	0.4342	-0.473	0.63643
140 Gy	-0.7035	0.4435	-1.586	0.11272
160 Gy	-1.3070	0.4942	-2.645	**0.00817**
180 Gy	-3.1884	0.6526	-4.886	**1..03e-06**
80 Gy	0.4089	0.4275	0.956	0.33882
No irradiation	2.1811	0.4208	5.183	**2.18e-07**

Fig 1. Irradiation effect on *D. melanogaster* male' sterility. The number of *D. melanogaster* adults emerged from fertile females and irradiated males. Different letters mean significant differences by Tukey Multiple Comparison tests ($p < 0.05$).

3.2. Irradiation effects on *D. melanogaster* male's longevity

In the longevity tests of *D. melanogaster* males, Kaplan-Meier curves showed significant differences in lifespan between the treatments (80 Gy, 100 Gy, 120 Gy, 140 Gy, 160 Gy, 180 Gy, the 'home control and 'trip control' conditions) (Mantel-Cox log-rank; $\chi 2 = 105.9$, d.f.= 7, P = < 2e-16) (Fig 2). The pairwise comparison test showed that control individuals have a higher probability of survival

than irradiated individuals. In particular, the ‘trip control’ condition showed significant differences with all the irradiation doses tested (80 Gy, $p = 0.039$; 100 Gy, $p = 0.027$; 120 Gy, $p = 0.013$; 140 Gy, $p = 0.017$; 160 Gy, $p < 0.001$; 180 Gy, $p < 0.001$; ‘home control’ condition, $p = 0.014$), with an average life of 71 days. Instead, the ‘home control’ condition showed significant differences only with 160 Gy ($p = 0.001$), 180 Gy ($p = 0.027$), and the ‘trip control’ condition, with an average life of 68 days. Significant differences were also observed in the longevity of males irradiated at 80 Gy and those irradiated at 160 Gy ($p < 0.001$), 180 Gy ($p < 0.001$), and the ‘trip control’ condition. The longevity of individuals irradiated at 160 Gy is significantly different from both control treatments and 80 Gy irradiation and also from 100 Gy ($p < 0.001$), 120 Gy ($p < 0.001$), 140 Gy ($p < 0.001$) and 180 Gy ($p = 0.013$). Furthermore, we found that the longevity of individuals irradiated at 180 Gy is significantly different from both control treatments, 80 Gy and 160 Gy, and also from 100 Gy ($p < 0.001$), 120 Gy ($p < 0.001$), and 140 Gy ($p < 0.001$). The average life of individuals irradiated at 160 Gy was 46 days and irradiated at 180 Gy was 55 days. We found no significant differences between the dose irradiations 80, 100, 120 and 140 Gy, with an average life of 63 days (Fig 2).

Fig 2. Irradiation effect on *D. melanogaster* male longevity. Kaplan-Meier curves showing the effect of different irradiation doses on the longevity of *D. melanogaster* males.

3.3. Irradiation effect on *D. melanogaster* male’ courtship behavior

In the “no-choice” conditions, the mean (± SE) courtship time widely ranged from 16.70% (± 3.98) (*D. melanogaster* irradiated at 160 Gy) up to 67.66% (± 8.30) (*D. suzukii* males) (Fig 3). The GLM model showed significant differences in the average courtship time among conditions (Table 3). Tukey's multiple comparison tests showed a significant difference between *D. suzukii* homospecific condition and the conditions with *D. melanogaster* irradiated at 160 Gy ($z = 4.346$, $p = <0.001$) and 180 Gy ($z = 3.552$, $p = 0.002$) and also a significant difference between *D. melanogaster* irradiated at 80 Gy and 160 Gy ($z = 2.697$, $p = 0.035$). No significant differences were observed in terms of

courtship time between *D. suzukii* males and *D. melanogaster* males irradiated at 80 Gy ($p > 0.05$) and between the courtship time of *D. melanogaster* irradiated at 80 and 180 Gy ($p > 0.05$) (Fig 3).

In the "choice" trials, the mean courtship times varied across treatments. In the conspecific condition with two *D. suzukii* males, the mean courtship time was 24.23% (± 6.17) for one male and 16% (± 3.50) for the other. In the heterospecific condition with irradiated *D. melanogaster* males at 80 Gy, the *D. suzukii* male exhibited a mean courtship time of 12.60% (± 3.06), while the *D. melanogaster* male displayed 31.83% (± 7.37). In the heterospecific condition with irradiated *D. melanogaster* males at 160 Gy, the *D. suzukii* male showed a mean courtship time of 24% (± 4.88), whereas the *D. melanogaster* male had 9.66% (± 4.61). In the heterospecific condition with *D. melanogaster* males irradiated at 180 Gy, the courtship time was 12.59% (± 1.94) for the *D. suzukii* male and 13.58% (± 5.68) for the *D. melanogaster* male (Fig 4).

The GLM analysis revealed significant differences in the average courtship time among conditions (Table 4), and Tukey's multiple comparisons test indicated a significant difference only between the courtship times of irradiated *D. melanogaster* males irradiated at 80 Gy and 160 Gy ($p = 0.016$; Fig 4). The Wilcoxon rank sum test highlighted significant differences only in the total courtship time between *D. melanogaster* males irradiated at 160 Gy and *D. suzukii* males ($W = 82.5$, $p = 0.002$) (Fig 4).

Table 3. Irradiation effect on *D. melanogaster* male' courtship time in "no-choice" condition. GLM model values are shown. Values in boldface indicate significant differences. *D. suz* = *Drosophila suzukii*; *D. mel* = *Drosophila melanogaster*.

**Fixed Effects**	**Estimate**	**±SE**	**z Value**	***p*-Value**
(Intercept)	2.8151	0.2273	12,384	**< 2e-16**
D. mel 180 Gy	0.2592	0.3204	0.809	0.41853
D. mel 80 Gy	0.8595	0.3188	2.697	**0.00069**

D. suz (homospecific condition)	1.3994	0.3220	4.346	**1.39e-05**

Table 4. Irradiation effect on *D. melanogaster* male' courtship time in "choice" condition. GLM model values are shown. Values in boldface indicate significant differences. *D. suz* = *Drosophila suzukii*; *D. mel* = *Drosophila melanogaster.*

**Fixed Effects**	**Estimate**	**±SE**	**z Value**	***p*-Value**
(Intercept)	3.4604	0.2447	14,139	**< 2e-16**
D. suz 80 Gy	-0.9271	0.3496	-2.652	**0.00799**
D. mel 160 Gy	-1.1924	0.3513	-3.394	**0.00068**
D. suz 160 Gy	-0.2828	0.3469	-0.815	0.41493
D. mel 180 Gy	-0.8515	0.3538	-2.407	**0.01609**
D. suz 180 Gy	-0.9279	0.3496	-2.654	**0.00794**
D. suz 1 (homospecific condition)	-0.6934	0.3530	-1.964	**0.04948**
D. suz 2 (homospecific condition)	-0.2730	0.3514	-0.777	0.43724

Fig 3. Courtship comparison in the "no-choice" trials. Courtship of *D. suzukii* males toward *D. suzukii* females (pink column); courtship of *D. melanogaster* males toward *D. suzukii* females at different irradiation doses (blue, green and orange columns). *** Tukey's multiple com-parison tests p < 0.001; ** Tukey's multiple comparison tests p < 0.01; * Tukey's multiple comparison tests p-value < 0.05. Black dots are box-plot outliers.

Fig 4. Courtship comparisons in "choice" trials. Time spent courting *D. suzukii* females by *D. suzukii* males and irradiated *D. melanogaster* males at 80, 160 and 180 Gy. *D. suz* = *D. suzukii* males (pink columns); *D. mel* 80 Gy = *D. melanogaster* males irradiated at 80 grey (blue column); *D. mel* 160 Gy = *D. melanogaster* males irradiated at 160 grey (green column); *D. mel* 180 Gy = *D. melanogaster* males irradiated at 180 grey (orange column). Black dots are box-plot outliers. * Tukey's multiple comparison tests p-value < 0.05; ** Wilcoxon rank sum test p < 0.01.

4. Discussion

Finding the best irradiation dose is a crucial ~~issue~~ that requires careful evaluation to develop a heterospecific SIT approach. We found that irradiation was highly effective at ~~reducing fertility~~. All irradiation doses led to a significant reduction in adult emergence with respect to the control condition (Table 1; Fig 1). We observed at the lower irradiation dose tested (80 Gy), only a 17% (± 5.38) average residual fertility that decreases as the irradiation doses increase until reaching 0.47 (± 0.34) average residual fertility at the highest dose tested (180 Gy) (Table 1). These results are consistent with previous findings. Studies about the effect of gamma rays on the sterility of *D. melanogaster* have been carried out in the 1960s and '70s. Henneberry (1963) (42) found that *D. melanogaster* irradiated males at 160 Gy dose produced nonviable eggs after mating with non-irradiated females. Accordingly, Nelson (1973) (43) observed that at 120 Gy 99.3% fewer progeny emerged than non-irradiated individuals.

A trade-off between the sterility and longevity of the irradiated males is critical in optimizing classic and heterospecific SIT applications (44). The survival analysis showed that irradiation significantly reduces the lifespan of *D. melanogaster* males, with the highest reductions in longevity at the highest doses. Control individuals lived on average for 70 days, whereas males irradiated at the highest doses (160–180 Gy) experienced a 50-day lifespan (Fig 2). This observation aligns with prior

studies indicating that irradiation-induced oxidative stress and cellular damage can impair physiological functions, shortening lifespan (45). Nelson et al. (1973) (43) also reported decreased longevity in irradiated *D. melanogaster*, with a similar reduction in lifespan at the highest dose tested of 150 Gy. The dose-dependent decrease in longevity must be carefully considered when applying SIT since male competitiveness may be compromised if they do not survive long enough to mate effectively in the wild. A reduction in the average lifespan of *D. melanogaster* from 70 in the controls to 50 days at the highest radiation doses can be seen unlike to compromise the effectiveness of SIT, as frequent releases of sterile individuals are typically part of the strategy. For instance, regarding screwworm *Cochliomyia hominivorax* (Coquerel), the releases have to occur weekly to maintain the critical ratio or even twice a week for the Mediterranean fruit fly and tsetse *Glossina austeni* (Wiedemann) (46–48). We want to highlight that this study was not addressed to assess the longevity of sterile individuals in field conditions, which can be lower than in protected field-cage situations, where sterile males have easy access to food and are protected from predation in the laboratory (49). This aspect certainly warrants further investigation.

The last part of this study was designed to assess the heterospecific courtship behavior of irradiated *D. melanogaster* males. A balance between sterility and behavioral competence when selecting an irradiation dose for pest control is critical. If males lose the ability to court females, their sterility will have limited impact on population suppression, as seen in studies of other insect species, such as the Queensland fruit fly (*Bactrocera tryoni* Froggatt), where high doses impaired sexual competitiveness (50). Our findings showed that irradiated *D. melanogaster* males retained their ability to court *D. suzukii* females even at the highest irradiation doses, suggesting that courtship behavior remains largely unaffected by irradiation. In the "no-choice condition", however, *D. melanogaster* males irradiated at 160 and 180 Gy exhibited significantly lower courtship activity compared to *D. suzukii* males toward conspecific females (Table 3; Fig 3). Conversely, under the "choice condition", *D. melanogaster* males courted *D. suzukii* females as much as *D. suzukii* males, even at the highest irradiation doses tested. Notably, *D. melanogaster* males irradiated at 160 Gy

showed reduced courtship toward *D. suzukii* females compared to *D. melanogaster* males irradiated at 80 Gy and *D. suzukii* males, corroborating the observations made in the “no-choice condition” (Fig 4). These results suggest two key points. First, the presence of *D. melanogaster* males seems to influence the courtship behavior of *D. suzukii* males, as they courted conspecific females more in the “no-choice condition” compared to the “choice condition”. The reduced courtship behavior observed at a radiation dose of 160 Gy suggests that higher doses may lead to behavioral impairments in *D. melanogaster* males. These impairments are likely attributable to physiological alterations or disruptions in neural circuits essential for mating displays. Ionizing radiation is known to damage neural pathways involved in courtship behavior, as evidenced in moths, where higher doses often result in physiological defects that reduce their competitiveness with wild populations (51,52). Radiation may also interfere with producing or expressing key biochemical and behavioral signals. During courtship, *D. melanogaster* males emit specific biochemical signals, such as cis-vaccenyl acetate, along with behavioral signals like wing vibrations and pheromone release, to stimulate female responses (53–55). Ionizing radiation may disrupt these signals, compromising the male's ability to communicate with females effectively. Similar disruptions have been observed in other pest species, such as *Callosobruchus chinensis* L. females and *Anthonomus grandis* (Boheman) males, where radiation-induced impairments in mating signals led to reduced courtship and mating success (56,57). Consequently, irradiated *D. melanogaster* males may fail to elicit appropriate responses from females, disrupting courtship and mating dynamics. In our study, while *D. melanogaster* males irradiated at 160 Gy showed reduced courtship, males exposed to 180 Gy courted *D. suzukii* females comparably to untreated *D. suzukii* males under both "no-choice" and "choice" conditions (Figs 3 and 4). Additionally, the highest courtship percentage was observed at the lowest tested dose of 80 Gy (Figs 3 and 4), supporting the notion that lower radiation doses may preserve male courtship behavior more effectively.

Overall, our study highlights the complex interactions between irradiation, longevity, sterility, and mating behavior in *D. melanogaster* and contributes to growing evidence of using heterospecific

SIT in pest control. Based on our results, the 80 Gy and 180 Gy radiation doses appear most suitable for further investigation. At 80 Gy, we observed the highest courtship rates and longest lifespan among the radiation doses tested. In comparison, at 180 Gy, we achieved the greatest reduction in fertility.

For an effective SIT program, it is essential that sterile males survive for a long time in their environment. Only in this way they can mate with a sufficient number of wild females and induce sterility in the population. If their quality is compromised and their longevity reduced, more frequent and larger-scale releases will be required to sustain a high overflooding ratio, ultimately increasing operational costs (58). Studies on *An. arabiensis* and *Ae. aegypti* have highlighted different factors influencing longevity. *An. arabiensis* was found to have a significantly shorter lifespan in field settings compared to laboratory conditions, whereas *Ae. aegypti* exhibited a stronger sensitivity to seasonality (59). Specifically, in a field experiment carried out in Vietnam, *Ae. aegypti* populations demonstrated significantly higher survival rates during the cool or hot dry seasons compared to the cool and wet seasons (60). Moreover, a synergistic effect of irradiation, packing, and chilling was observed to compromise the longevity of *An. arabiensis* —an effect that was not detected in *Ae. aegypti* (59). These results suggest that the impact of irradiation on lifespan is highly species-dependent. In our case, it is essential first to assess the differences in *D. melanogaster* males' longevity kept under laboratory conditions and those exposed to environmental conditions. This would allow us to better evaluate the lifespan reduction at 180 Gy and determine whether it constitutes a limitation for SIT applications. Conversely, a higher residual fertility at 80 Gy than 180 Gy might be less restrictive given that we are not dealing with a pest or invasive species. Unlike in standard SIT applications for pest control, achieving the high sterility levels required to release pest males (as discussed by Bakri et al. 2021 (61) and Parker et al. 2021 (62)) may not be necessary in this context. Based on these considerations, the present study has demonstrated that an irradiation dose of 80 Gy seems to be more effective. However, further studies are needed better to evaluate both longevity and mating performance under field conditions.

Greenhouses and other enclosed environments appear ideal for implementing the heterospecific Sterile Insect Technique (h-SIT) to manage *D. suzukii* populations. Studies on plastic- and mesh-covered tunnels have shown that mechanical barriers alone can significantly reduce *D. suzukii* populations in these confined areas. This reduction is due not only to the physical exclusion provided by the barriers but also to creating an unfavorable microclimate for the pest's survival (63). Although complete exclusion cannot be achieved through mechanical barriers alone, integrating h-SIT with these measures could enhance the overall effectiveness of biocontrol strategies.

Conclusions

Our findings highlight the critical balance between sterility, longevity, and mating behavior in *D. melanogaster* for heterospecific SIT applications. Among the tested doses, 80 Gy emerged as the most effective, preserving male longevity and mating performance while significantly reducing fertility. While 180 Gy achieved the highest sterility, the potential lifespan and courtship behavior trade-offs warrant further evaluation. Future studies should focus on-field performance to refine SIT protocols. Integrating h-SIT with mechanical barriers in controlled environments like greenhouses could enhance *D. suzukii* management, making 80 Gy a promising dose for practical implementation.

Acknowledgments

We thank Alessandra Spanò, Elisa Michelangeli and Giulia Pezzi for their technical help.

References

1. Cini A, Ioriatti C, Anfora G. A review of the invasion of *Drosophila suzukii* in Europe and a draft research agenda for integrated pest management. 2012 [cited 2025 Jan 21]; Available from: https://openpub.fmach.it/handle/10449/21029

2. Cini A, Anfora G, Escudero-Colomar LA, Grassi A, Santosuosso U, Seljak G, et al. Tracking the invasion of the alien fruit pest *Drosophila suzukii* in Europe. J Pest Sci. 2014 Dec;87(4):559–66.

3. Asplen MK, Anfora G, Biondi A, Choi DS, Chu D, Daane KM, et al. Invasion biology of spotted wing Drosophila (*Drosophila suzukii*): a global perspective and future priorities. J Pest Sci. 2015 Sep;88(3):469–94.

4. Roy AS. Revision of the EPPO datasheets: a successful project. EPPO Bulletin. 2024;54(3):345–54.

5. De Ros G. The Economic Analyses of the *Drosophila suzukii*'s Invasions: A Mini-review. Neotrop Entomol. 2024 Apr 1;53(2):244–53.

6. Farnsworth D, Hamby KA, Bolda M, Goodhue RE, Williams JC, Zalom FG. Economic analysis of revenue losses and control costs associated with the spotted wing drosophila, *Drosophila suzukii* (Matsumura), in the California raspberry industry. Pest Management Science. 2017;73(6):1083–90.

7. Rota-Stabelli O, Blaxter M, Anfora G. *Drosophila suzukii*. Current Biology. 2013 Jan 7;23(1):R8–9.

8. Walsh DB, Bolda MP, Goodhue RE, Dreves AJ, Lee J, Bruck DJ, et al. *Drosophila suzukii* (Diptera: Drosophilidae): Invasive Pest of Ripening Soft Fruit Expanding its Geographic Range and Damage Potential. Journal of Integrated Pest Management. 2011 Apr 1;2(1):G1–7.

9. Bruck DJ, Bolda M, Tanigoshi L, Klick J, Kleiber J, DeFrancesco J, et al. Laboratory and field comparisons of insecticides to reduce infestation of *Drosophila suzukii* in berry crops: Controlling *D. suzukii* in berries. Pest Manag Sci. 2011 Nov;67(11):1375–85.

10. Van Timmeren S, Isaacs R. Control of spotted wing drosophila, *Drosophila suzukii*, by specific insecticides and by conventional and organic crop protection programs. Crop Protection. 2013 Dec 1;54:126–33.

11. Diepenbrock LM, Rosensteel DO, Hardin JA, Sial AA, Burrack HJ. Season-long programs for control of *Drosophila suzukii* in southeastern U.S. blueberries. Crop Protection. 2016 Mar 1;81:76–84.

12. Tabuloc CA, Carlson CR, Ganjisaffar F, Truong CC, Chen CH, Lewald KM, et al. Transcriptome analysis of *Drosophila suzukii* reveals molecular mechanisms conferring pyrethroid and spinosad resistance. Sci Rep. 2024 Aug 27;14(1):19867.

13. Schetelig MF, Lee KZ, Otto S, Talmann L, Stökl J, Degenkolb T, et al. Environmentally sustainable pest control options for *Drosophila suzukii*. Journal of Applied Entomology. 2018;142(1–2):3–17.

14. Cha DH, Hesler SP, Park S, Adams TB, Zack RS, Rogg H, et al. Simpler is better: fewer non-target insects trapped with a four-component chemical lure vs. a chemically more complex food-type bait for rosophila suzukii. Entomologia Experimentalis et Applicata. 2015;154(3):251–60.

15. Burrack HJ, Asplen M, Bahder L, Collins J, Drummond FA, Guédot C, et al. Multistate Comparison of Attractants for Monitoring *Drosophila suzukii* (Diptera: Drosophilidae) in Blueberries and Caneberries. Environmental Entomology. 2015 Jun 1;44(3):704–12.

16. Rossi Stacconi MV, Grassi A, Ioriatti C, Anfora G. Augmentative releases of Trichopria drosophilae for the suppression of early season *Drosophila suzukii* populations. BioControl. 2019 Feb 1;64(1):9–19.

17. McInnis DO, Rendon P, Jang E, Van Sauers-Muller A, Sugayama R, Malavasi A. Interspecific Mating of Introduced, Sterile *Bactrocera dorsalis* with Wild *B. carambolae* (Diptera: Tephritidae) in Suriname: a Potential Case for Cross-Species Sterile Insect Technique. Annals of the Entomological Society of America. 1999 Sep 1;92(5):758–65.

18. Honma A, Kumano N, Noriyuki S. Killing two bugs with one stone: a perspective for targeting multiple pest species by incorporating reproductive interference into sterile insect technique: Reproductive interference and SIT. Pest Manag Sci. 2019 Mar;75(3):571–7.

19. Cerasti F, Mastrantonio V, Dallai R, Cristofaro M, Porretta D. Applying Satyrization to Insect Pest Control: The Case of the Spotted Wing Drosophila, *Drosophila suzukii* Matsumura. Insects. 2023 Jun 19;14(6):569.

20. Cerasti F, Cristofaro M, Mastrantonio V, Scifo J, Verna A, Canestrelli D, et al. Can reproductive interference be integrated into the Sterile Insect Technique for pest control? Insights from the spotted wing fly *Drosophila suzukii*. bioRxiv. 2024 Jan 1;2024.09.05.611447.

21. Bourtzis K, Vreysen MJB. Sterile Insect Technique (SIT) and Its Applications. Insects. 2021 Jul 13;12(7):638.

22. Klassen W. Area-Wide Integrated Pest Management and the Sterile Insect Technique. In: Dyck VA, Hendrichs J, Robinson AS, editors. Sterile Insect Technique: Principles and Practice in Area-Wide Integrated Pest Management. Dordrecht: Springer Netherlands; 2005 [cited 2025 Jan 28]. p. 39–68. Available from: https://doi.org/10.1007/1-4020-4051-2_2

23. Ribeiro JMC. Can Satyrs Control Pests and Vectors? Journal of Medical Entomology. 1988 Nov 1;25(6):431–40.

24. Gröning J, Hochkirch A. Reproductive Interference Between Animal Species. The Quarterly Review of Biology. 2008 Sep;83(3):257–82.

25. Shuker DM, Burdfield-Steel ER. Reproductive interference in insects: Reproductive interference in insects. Ecol Entomol. 2017 Aug;42:65–75.

26. Kyogoku D. Reproductive interference: ecological and evolutionary consequences of interspecific promiscuity. Popul Ecol. 2015 Apr;57(2):253–60.

27. Mitchell C, Leigh S, Alphey L, Haerty W, Chapman T. Reproductive interference and Satyrisation: mechanisms, outcomes and potential use for insect control. J Pest Sci. 2022 Jun;95(3):1023–36.

28. Guerfali MM, Parker A, Fadhl S, Hemdane H, Raies A, Chevrier C. Fitness and Reproductive Potential of Irradiated Mass-Reared Mediterranean Fruit Fly Males *Ceratitis capitata* (Diptera: Tephritidae): Lowering Radiation Doses. flen. 2011 Dec;94(4):1042–50.

29. Helinski ME, Parker AG, Knols BG. Radiation-induced sterility for pupal and adult stages of the malaria mosquito *Anopheles arabiensis*. Malar J. 2006 Dec;5(1):41.

30. Helinski MEH, Knols BGJ. Mating Competitiveness of Male *Anopheles arabiensis* Mosquitoes Irradiated with a Partially or Fully Sterilizing Dose in Small and Large Laboratory Cages. Journal of Medical Entomology. 2008 Jul 1;45(4):698–705.

31. Bond JG, Osorio AR, Avila N, Gómez-Simuta Y, Marina CF, Fernández-Salas I, et al. Optimization of irradiation dose to *Aedes aegypti* and *Ae. albopictus* in a sterile insect technique program. PLOS ONE. 2019 Feb 19;14(2):e0212520.

32. Kittayapong P, Ninphanomchai S, Thayanukul P, Yongyai J, Limohpasmanee W. Comparison on the quality of sterile *Aedes aegypti* mosquitoes produced by either radiation-based sterile insect technique or Wolbachia-induced incompatible insect technique. PLOS ONE. 2025 Feb 12;20(2):e0314683.

33. Silva-Soares NF, Nogueira-Alves A, Beldade P, Mirth CK. Adaptation to new nutritional environments: larval performance, foraging decisions, and adult oviposition choices in *Drosophila suzukii*. BMC Ecol. 2017 Dec;17(1):21.

34. Baccaro S, Cemmi A, Di Sarcina I, Ferrara G. S. Baccaro, A. Cemmi, I. Di Sarcina, G. Ferrara. Gamma irradiation Calliope facility at ENEA–Casaccia Research Centre (Rome, Italy). ENEA Technical Report RT/2019/4/ENEA, ISSN/0393-3016.

35. Lasbleiz C, Ferveur JF, Everaerts C. Courtship behaviour of *Drosophila melanogaster* revisited. Animal Behaviour. 2006 Nov;72(5):1001–12.

36. Revadi S, Lebreton S, Witzgall P, Anfora G, Dekker T, Becher P. Sexual Behavior of *Drosophila suzukii*. Insects. 2015 Mar 9;6(1):183–96.

37. Hothorn T, Bretz F, Westfall P. Simultaneous Inference in General Parametric Models. Biometrical J. 2008 Jun;50(3):346–63.

38. Kaplan EL, Meier P. Nonparametric Estimation from Incomplete Observations. Journal of the American Statistical Association. 1958 Jun;53(282):457–81.

39. Alboukadel K, Marcin K, Przemyslaw B. survminer: Drawing Survival Curves using "ggplot2." CRAN: Contributed Packages [Internet]. 2016 Jan [cited 2025 Jan 28]; Available from: https://cir.nii.ac.jp/crid/1360300776601012224

40. Wickham H, Averick M, Bryan J, Chang W, McGowan LD, François R, et al. Welcome to the Tidyverse. Journal of Open Source Software. 2019 Nov 21;4(43):1686.

41. Team RC. R A language and environment for statistical computing, R Foundation for Statistical. Computing. 2020 [cited 2025 Jan 18]. Available from: https://cir.nii.ac.jp/crid/1370298755636824325

42. Henneberry TJ. Effects of Gamma Radiation on the Fertility and Longevity of *Drosophila melanogaster*. Journal of Economic Entomology. 1963 Jun 1;56(3):279–81.

43. Nelson FRS. *Drosophila melanogaster*: Effects of Gamma Radiation on Fecundity and Longevity1. Journal of Economic Entomology. 1973 Feb 1;66(1):257–8.

44. Hendrichs J, Pereira R, Vreysen MJB. Area-Wide Integrated Pest Management: Development and Field Application. 1st ed. Boca Raton: CRC Press; 2021 [cited 2022 Dec 23]. Available from: https://www.taylorfrancis.com/books/9781003169239

45. Bakri A, Heather N, Hendrichs J, Ferris I. Fifty Years of Radiation Biology in Entomology: Lessons Learned from IDIDAS. Annals of the Entomological Society of America. 2005 Jan 1;98(1):1–12.

46. Tan KH. Area-wide control of fruit flies and other insect pests: joint proceedings of the International conference on area-wide control of insect pests, May 28-June 2, 1998 and the Fith international symposium on fruit flies of economic importance, June 1-5, 1998, Penang, Malaysia. Pulau Pinang: Penerbi USM; 2000.

47. Vreysen MJB, Saleh KM, Ali MY, Abdulla AM, Zhu ZR, Juma KG, et al. *Glossina austeni* (Diptera: Glossinidae) Eradicated on the Island of Unguja, Zanzibar, Using the Sterile Insect Technique. Journal of Economic Entomology. 2000 Feb 1;93(1):123–35.

48. Baumhover AH. A PERSONAL ACCOUNT OF DEVELOPING THE STERILE INSECT TECHNIQUE TO ERADICATE THE SCREWWORM FROM CURACAO, FLORIDA AND THE SOUTHEASTERN UNITED STATES. flen. 2002 Dec;85(4):666–73.

49. Hendrichs J, Robinson AS, Cayol JP, Enkerlin W. MEDFLY AREAWIDE STERILE INSECT TECHNIQUE PROGRAMMES FOR PREVENTION, SUPPRESSION OR ERADICATION: THE IMPORTANCE OF MATING BEHAVIOR STUDIES. Florida Entomologist. 2002 Mar;85(1):1–13.

50. Fanson B g., Sundaralingam S, Jiang L, Dominiak B c., D'Arcy G. A review of 16 years of quality control parameters at a mass-rearing facility producing Queensland fruit fly, *Bactrocera tryoni*. Entomologia Experimentalis et Applicata. 2014;151(2):152–9.

51. Marec F, Vreysen MJB. Advances and Challenges of Using the Sterile Insect Technique for the Management of Pest Lepidoptera. Insects. 2019 Nov;10(11):371.

52. LaChance LE, Graham CK. Insect radiosensitivity: Dose curves and dose-fractionation studies of dominant lethal mutations in the mature sperm of 4 insect species. Mutation Research/Fundamental and Molecular Mechanisms of Mutagenesis. 1984 Jun;127(1):49–59.

53. Antony C, Jallon JM. The chemical basis for sex recognition in *Drosophila melanogaster*. Journal of Insect Physiology. 1982 Jan 1;28(10):873–80.

54. Fernández MP, Kravitz EA. Aggression and Courtship in Drosophila: Pheromonal Communication and Sex Recognition. J Comp Physiol A Neuroethol Sens Neural Behav Physiol. 2013 Nov;199(11):10.1007/s00359-013-0851–5.

55. Ejima A. Pleiotropic actions of the male pheromone cis-vaccenyl acetate in *Drosophila melanogaster*. J Comp Physiol A Neuroethol Sens Neural Behav Physiol. 2015;201(9):927–32.

56. Earle NW, Simmons LA, Nilakhe SS, Villavaso EJ, McKibben GH, Sikorowski P. Pheromone Production and Sterility in Boll Weevils: Effect of Acute and Fractionated Gamma Irradiation134. Journal of Economic Entomology. 1978 Aug 1;71(4):591–5.

57. Chiluwal K, Kim J, Bae SD, Roh GH, Park HJ, Park CG. Effect of Gamma Irradiation on Fecundity, Sterility, and Female Sex Pheromone Production of *Callosobruchus chinensis* (Coleoptera: Bruchidae). J Econ Entomol. 2019 Feb 12;112(1):156–63.

58. Lance DR, McInnis DO. Biological Basis of the Sterile Insect Technique. In: Sterile Insect Technique. 2nd ed. CRC Press; 2021.

59. Culbert NJ, Maiga H, Somda NSB, Gilles JRL, Bouyer J, Mamai W. Longevity of mass-reared, irradiated and packed male *Anopheles arabiensis* and *Aedes aegypti* under simulated environmental field conditions. Parasit Vectors. 2018 Nov 21;11:603.

60. Hugo LE, Jeffery JAL, Trewin BJ, Wockner LF, Yen NT, Le NH, et al. Adult Survivorship of the Dengue Mosquito *Aedes aegypti* Varies Seasonally in Central Vietnam. PLOS Neglected Tropical Diseases. 2014 Feb 13;8(2):e2669.

61. Bakri A, Mehta K, Lance DR. Sterilizing Insects with Ionizing Radiation. In: Sterile Insect Technique. 2nd ed. CRC Press; 2021.

62. Parker AG, Vreysen MJB, Bouyer J, Calkins CO. Sterile Insect Quality Control/Assurance. In: Sterile Insect Technique. 2nd ed. CRC Press; 2021.

63. Rogers MA, Burkness EC, Hutchison WD. Evaluation of high tunnels for management of *Drosophila suzukii* in fall-bearing red raspberries: Potential for reducing insecticide use. J Pest Sci. 2016 Jul 1;89(3):815–21.

Figure 1

Click here to access/download;Figure;fig1.tif

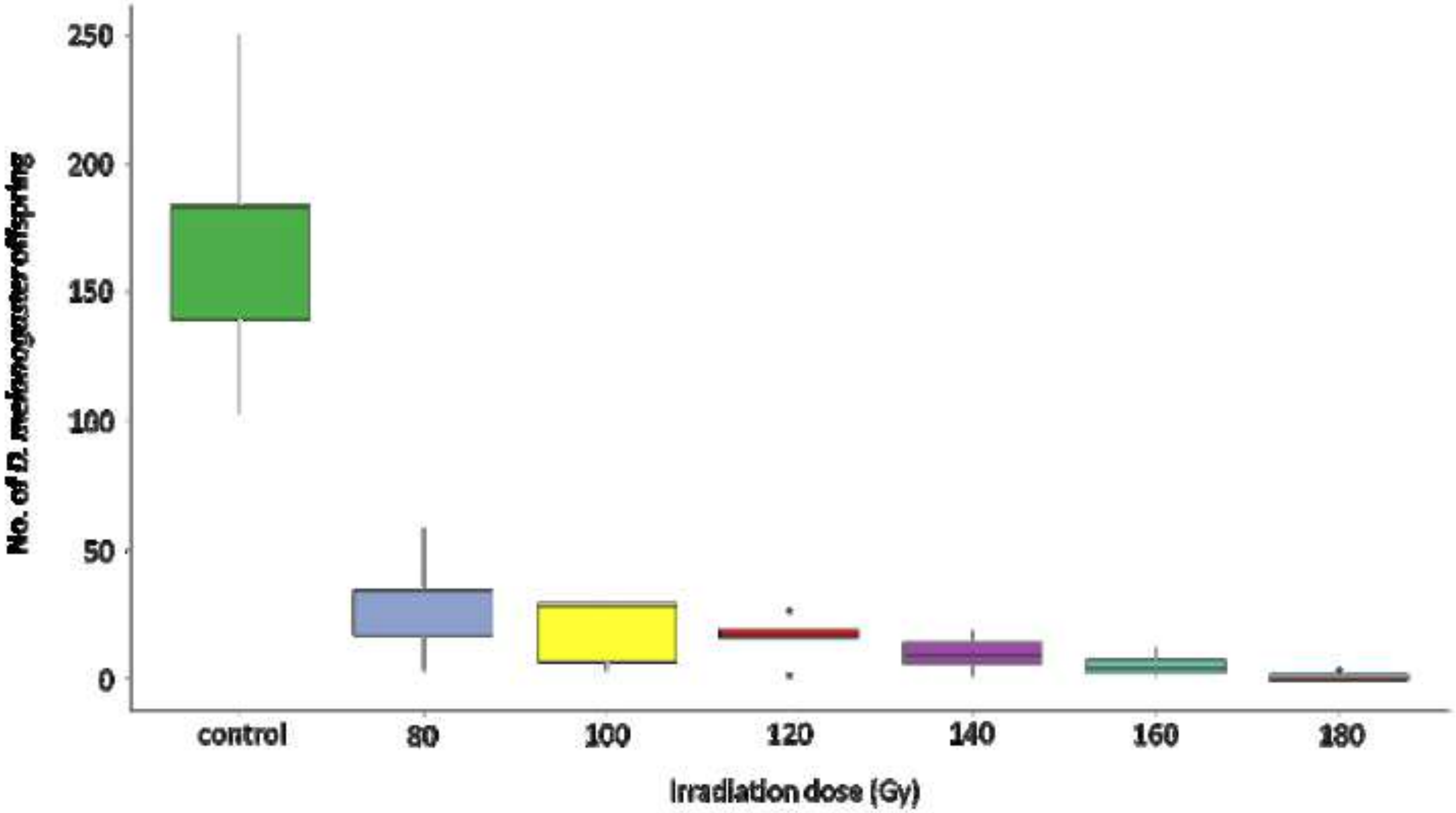

Figure 2

Click here to access/download;Figure;fig2.tif

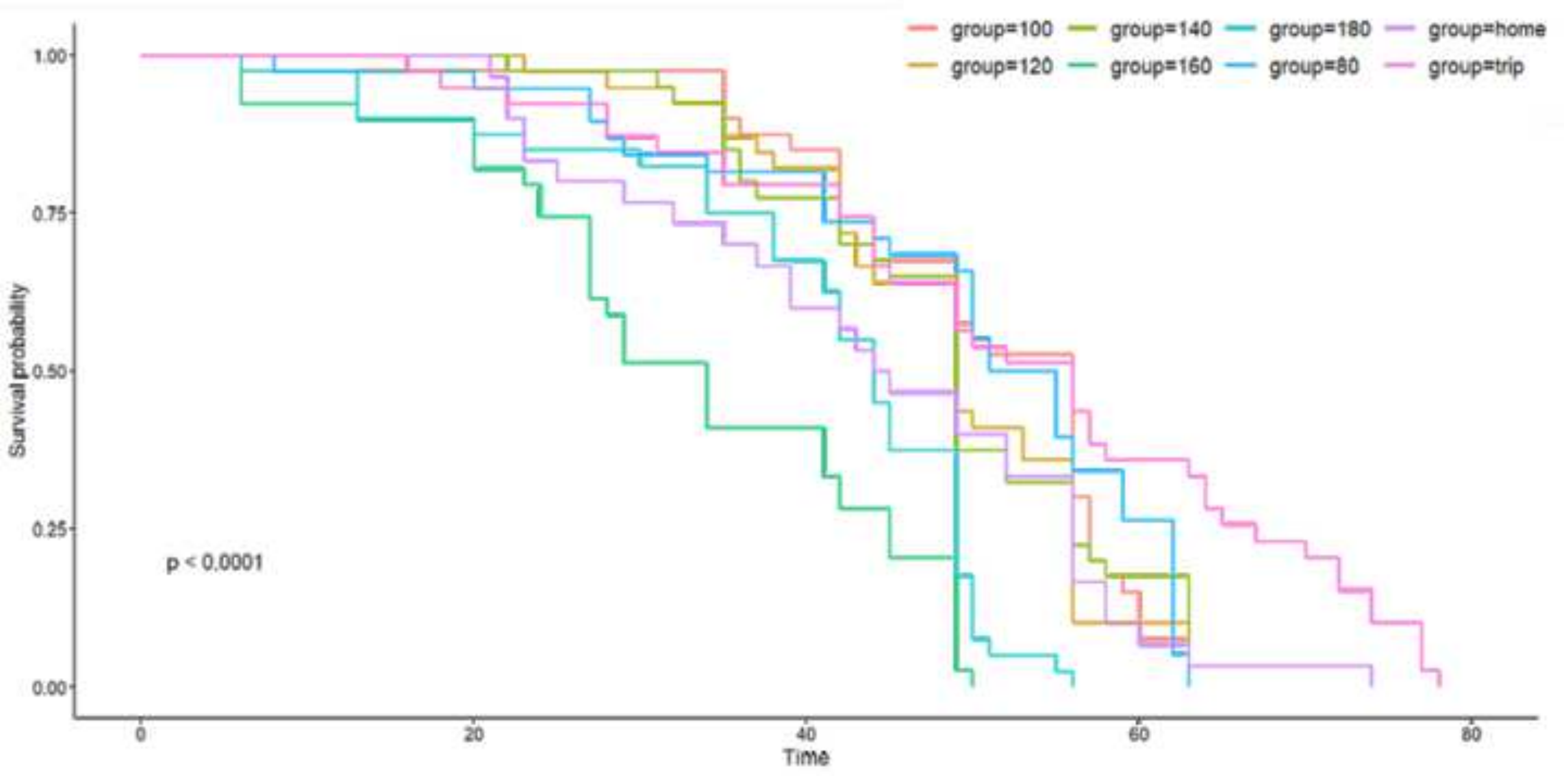

Figure 3
Click here to access/download;Figure;fig3.tif

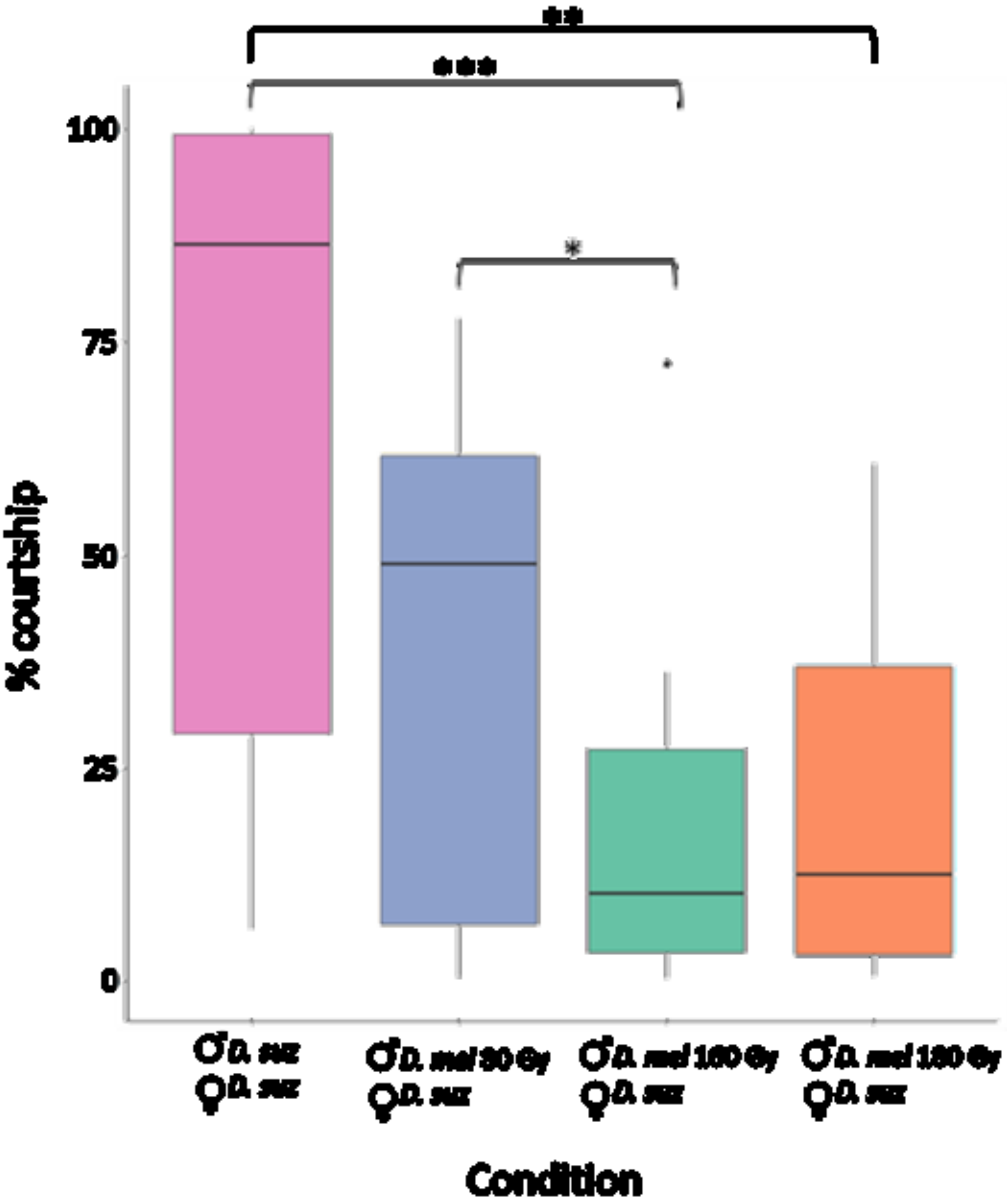

Click here to access/download;Figure;fig4.tif

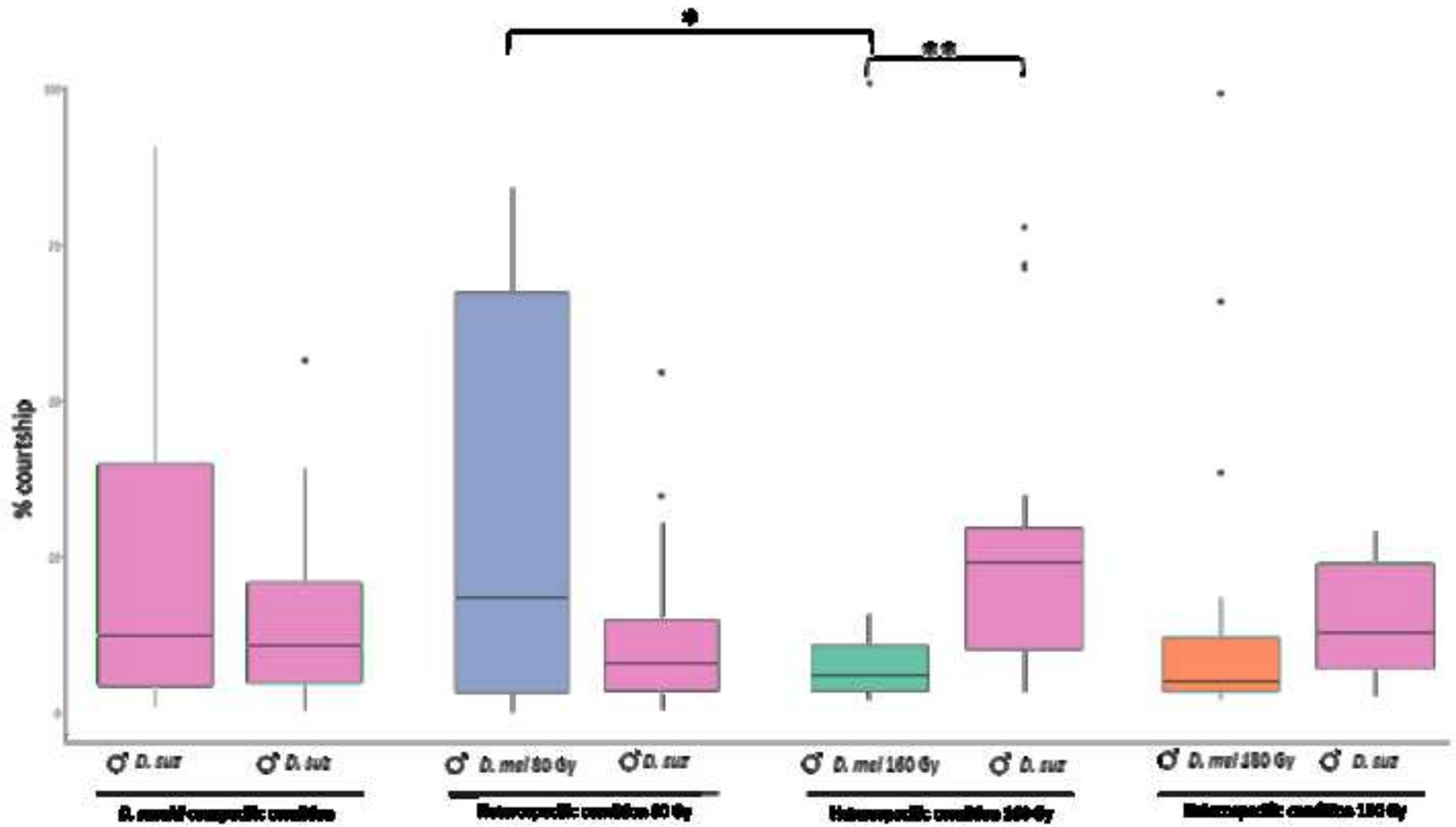

Supplement: Attachment — Submitted filename: PONE-D-25-09324.pdf [file pone.0320546.s001.pdf]
