# Supplementary material for: Optimizing irradiation dose for Drosophila melanogaster males to enhance heterospecific Sterile Insect Technique (h-SIT) against Drosophila suzukii
Source: PLoS One. 2025 Jul 3;20(7):e0320546. doi: 10.1371/journal.pone.0320546 (PMC12225833; doi:10.1371/journal.pone.0320546)
Supplement: Attachment — Submitted filename: Response to Reviewers.docx [file pone.0320546.s002.docx]

**Response to the reviewer's and Editor's comments**

To the Referees and Editor,

We were pleased to receive your comments for improving our study, and we thank you.

Editor’s comments

**Editor:** 1. Please ensure that your manuscript meets PLOS ONE's style requirements, including those for file naming.

**Authors:** We thank the editor for the comments. We checked that our manuscript meets PLOS ONE's style requirements.

**Editor:** 2. In the online submission form, you indicated that raw data will be available from the authors under request. All PLOS journals now require all data underlying the findings described in their manuscript to be freely available to other researchers, either 1. In a public repository, 2. Within the manuscript itself, or 3. Uploaded as supplementary information.

This policy applies to all data except where public deposition would breach compliance with the protocol approved by your research ethics board. If your data cannot be made publicly available for ethical or legal reasons (e.g., public availability would compromise patient privacy), please explain your reasons on resubmission and your exemption request will be escalated for approval.

**Authors:** We modified the information about raw data and uploaded the dataset to a public repository (Raw data are now available from Figshare: 10.6084/m9.figshare.28777601).

**Editor:** 3. Please review your reference list to ensure that it is complete and correct. If you have cited papers that have been retracted, please include the rationale for doing so in the manuscript text, or remove these references and replace them with relevant current references. Any changes to the reference list should be mentioned in the rebuttal letter that accompanies your revised manuscript. If you need to cite a retracted article, indicate the article’s retracted status in the References list and also include a citation and full reference for the retraction notice.

**Authors:** We checked that the reference list was complete and correct. We modified only the Reference 20 following the Editor suggestion (see comments below).

Additional editor comments:

**Editor**: 1) The quality of the images in the Figures needs to be improved (also pick less closely related colors for the lines Fig2).

**Authors:** We improved the quality of the images in the Figures and pick less closely related colors for the lines in Figure 2.

**Editor:** 2) Reference 20 has a different title than the apparent BIORXIV preprint match 'Developing heterospecific Sterile Insect Technique for pest control: insights from the spotted wing fly Drosophila suzukii'. Please explain. What is the status of this manuscript as well as its relationship to this submission? Please confirm that there is no overlap in the data.

**Authors:** We apologize for the typo, which has been corrected in the revised version of the manuscript. The correct title is 'Developing heterospecific Sterile Insect Technique for pest control: insights from the spotted wing fly *Drosophila suzukii'*. The incorrect title referred to an earlier version of the preprint, which was subsequently updated following the revisions suggested by the journal to which we submitted that article. We confirm no overlap in the data since the two articles have completely different data sets.

**Editor:** 3) You will need to fix your data availability statement. Making raw data only available upon request is not compatible with your answer 'Yes' to the data availability question. You can use supplements or a public data repository to fix this or answer 'No' to the question and provide acceptable justification.

**Authors:** We fixed our data availability statement. Raw data are now available from Figshare: 10.6084/m9.figshare.28777601.

**Referee: 1**

**Reviewer:** The study focuses on optimizing the irradiation dose for *Drosophila melanogaster* males to achieve maximum sterility while minimizing the impact on insect fitness. The authors attempt to correlate these findings with their previous research on the Heterospecific Sterile Insect Technique (hSIT) against *Drosophila suzukii*. However, no clear relationship between the current study and their previous hSIT findings was established. If an integrated approach combining hSIT and irradiation is required for managing *D. suzukii*, the experiments should optimize irradiation doses following heterospecific mating. This would allow for a comprehensive evaluation of key parameters, including fertility, sterility, fitness, and courtship behaviors.

**Authors:** We thank the reviewer for this comment. In our previous studies we investigated reproductive interference between un-irradiated *D. melanogaster* males and *D. suzukii* females, obtaining promising results (Cerasti et al., 2023). For this reason, our research has progressed toward evaluating the reproductive interference caused by irradiated *D. melanogaster* males, bringing us closer to a potential SIT-based application. We included this information in the revised version of the manuscript (lines 110-115 of the tracking version of the manuscript).

Cerasti F, Mastrantonio V, Dallai R, Cristofaro M, Porretta D. Applying Satyrization to Insect Pest Control: The Case of the Spotted Wing Drosophila, *Drosophila suzukii* Matsumura. Insects. 2023 Jun 19;14(6):569.

**Reviewer:** If fitness is compromised in *D. melanogaster* males during heterospecific mating (line 68-72) with *D. suzukii* females, the males are already weakened before undergoing irradiation. Thus, D. melanogaster males should undergo irradiation after heterospecific mating rather than using untreated males for a more accurate assessment of cumulative fitness trade-offs.

**Authors:** The sentence in lines 68-72 is a general description of how reproductive interference acts. The fitness cost we refer to does not affect *D. melanogaster* males, but rather the *D. suzukii* species, as confirmed by our previous studies, which investigated reproductive interference between the two species without exposing *D. melanogaster* males to irradiation (Cerasti et al., 2023).

Cerasti F, Mastrantonio V, Dallai R, Cristofaro M, Porretta D. Applying Satyrization to Insect Pest Control: The Case of the Spotted Wing Drosophila, *Drosophila suzukii* Matsumura. Insects. 2023 Jun 19;14(6):569.

**Reviewer:** In my view, the current findings cannot be directly correlated with previous hSIT experiments. Below are my main suggestions to enhance the study's impact: 1. Title Revision: The title should be rephrased for clarity. I suggest the following: "Optimizing Irradiation Dose for *Drosophila melanogaster* Males to Enhance Heterospecific Sterile Insect Technique Against *Drosophila suzukii*."

**Author:** We agree with the title suggestion. We modified the title of the manuscript.

**Reviewer:** 2. Clarify the Study's Importance: If hSIT alone results in complete sterility, why is an optimized irradiation dose necessary? Please address the significance of this study in both the abstract and introduction sections.

**Authors:** We thank the reviewer for the comment. In this h-SIT the sterility in the target pest population is ensured by the reproductive post-zygotic isolation mechanisms between the released *D. melanogaster* males and the wild *D. suzukii* females. Male sterilization avoids potential adverse environmental effects in the natural population dynamics of *D. melanogaster*. As suggested, we addressed it in the abstract (lines 26-29 of the tracking version of the manuscript) and introduction (lines 115-119 of the tracking version of the manuscript) sections.

**Reviewer:** 3. Expand on Fitness Costs (Introduction, Line 68): The authors mention that hSIT results in fitness costs for one or both interacting individuals. Please elaborate on how these fitness costs manifest and identify which traits are affected. If flight ability is compromised, it could reduce the feasibility of field releases.

**Authors:** Thank you for the suggestion. Reproductive interference is a heterospecific interaction that can result in a fitness cost for one or both species involved. In general, the fitness costs are directly tied to behaviors or outcomes related to mating, fertilization, or reproductive success, and flight ability or general physiological performance are not considered direct targets of RI costs. We highlighted this issue in the revised version of the paper and included key references (lines 90-92 of the tracking version of the manuscript).

**Reviewer:** 4. Quantify Offspring Reduction (Line 87): Please specify the level of offspring reduction achieved in your previous hSIT studies against *D. suzukii*. If 100% sterility was not achieved through hSIT alone, highlight how this justifies the need for irradiation to enhance hSIT's effectiveness.

**Authors:** The mating between *D. melanogaster* males (irradiated and un-irradiated) and *D. suzukii* females leads to 100% sterility as there is complete post-zygotic isolation. The sentence: “Furthermore, under laboratory conditions, *D. melanogaster* males irradiated at 60 and 80 Gy were able to court and mate with *D. suzukii* females, leading to a significant reduction in *D. suzukii* offspring.” refers to our previous study where we investigated whether reproductive interactions between *D. melanogaster* males and *D. suzukii* individuals incur fitness costs for *D. suzukii* females. We observed a significant effect of *D. melanogaster* males on the *D. suzukii* offspring. In fact, the mean number of the individuals originating from five pairs of *D. suzukii* was 37.95 (± 3.62) (± standard error) in the control tests without the presence of *D. melanogaster* males, while it was 16.11 (± 5.58) and 13.89 (±3.69), when 40 and 60 irradiated *D. melanogaster* males were added, respectively (Cerasti et al., 2025). The sentence “However, the induced sterility in *D. melanogaster* males was not complete at these doses” refers to the sterility experiment where we tested the induced sterility in *D. melanogaster* males irradiated at 60 and 80 Gy mated with un-irradiated *D. melanogaster* females. At these two doses, we did not achieve 100% sterility in males (Cerasti et al., 2025). For this reason, in the current study under revision, we tested whether higher irradiation doses could lead to higher male sterility without reducing male performance.

Cerasti F, Cristofaro M, Mastrantonio V, Scifo J, Verna A, Canestrelli D, et al. Developing heterospecific Sterile Insect Technique for pest control: insights from the spotted wing fly Drosophila suzukii [Internet]. bioRxiv; 2025 [cited 2025 Feb 20]. p. 2024.09.05.611447. Available from: https://www.biorxiv.org/content/10.1101/2024.09.05.611447v2

**Reviewer:** 5. Clarify Behavioral Analysis: Since courtship behavior is complex, and you specifically analyze the time males spend with females, it would be more precise to state: "We analyzed the time spent by male *D. melanogaster* with *D. suzukii* females" rather than using the broader term "courtship behavior.

**Authors:** We thank the Reviewer for this comment. We agree with the reviewer that courtship behavior encompasses complex interactions. In our study, we specifically focused only on the time spent by males courting *D. suzukii* females. For this reason, we modified the text following your suggestion (lines 130-132; 199; 325 of the tracking version of the manuscript).

**Referee 2**

**Reviewer:** The science and experimental design is appropriate for the research questions being addressed. However, there is a need to improve on the presentation of information including methods, results and discussion. The major concern is on the writing style/English. I have detailed specific concern in the main manuscript submission document.

**Authors:** We thank the Reviewer for the work done on the manuscript. The Reviewer detailed specific concern in the main manuscript submission document, and we included all changes in the revised version of the paper (see tracking version of the manuscript).
