# Supplementary material for: Optimizing irradiation dose for Drosophila melanogaster males to enhance heterospecific Sterile Insect Technique (h-SIT) against Drosophila suzukii
Source: PLoS One. 2025 Jul 3;20(7):e0320546. doi: 10.1371/journal.pone.0320546 (PMC12225833; doi:10.1371/journal.pone.0320546)
Supplement: Attachment — Submitted filename: Response_to_Reviewers_auresp_2.docx [file pone.0320546.s003.docx]

**Response to the reviewer's and Editor's comments**

We sincerely thank the Academic Editor and the reviewers for their thorough evaluation and constructive feedback throughout the review process. We are pleased that the revised version of our manuscript was well received.

As requested by the Academic Editor, we have revised the figures by increasing font sizes to enhance readability without affecting the overall layout. All figure files have been updated accordingly. Apart from these changes, we made a single minor correction to a word in the main text for clarity.

We hope that the revised version meets the final requirements for publication in *PLOS ONE*.

Sincerely,

Flavia Cerasti, Ph.D.

Corresponding Author

Department of Environmental Biology, Sapienza University of Rome
